# Patterns of nutrients’ intake at six months in the northeast of Italy: a cohort study

**DOI:** 10.1186/1471-2431-14-127

**Published:** 2014-05-22

**Authors:** Paola Pani, Claudia Carletti, Alessandra Knowles, Maria Parpinel, Federica Concina, Marcella Montico, Adriano Cattaneo

**Affiliations:** 1 Health Services Research and International Health, Institute for Maternal and Child Health IRCCS Burlo Garofolo, Via dell’Istria 65/1, 34137 Trieste, Italy; 2 Department of Medical and Biological Sciences, University of Udine, Udine, Italy; 3 Epidemiology and Biostatistics, Institute for Maternal and Child Health IRCCS Burlo Garofolo, Trieste, Italy

**Keywords:** Breastfeeding, Complementary feeding, Food and nutrient intake, Public health, Policy, Italy

## Abstract

**Background:**

Adequate complementary feeding is recognized as an important predictor of health later in life. The objective of this study was to describe the feeding practices and nutrients’ intake, and their association with breastfeeding at six months of age, in a cohort of infants enrolled at birth in the maternity hospital of Trieste, Italy.

**Methods:**

Out of 400 infants enrolled at birth, 268 (67%) had complete data gathered through a 24-hour feeding diary on three separate days at six months, and two questionnaires administered at birth and at six months. Data from feeding diaries were used to estimate nutrients’ intakes using the Italian food composition database included in the software. To estimate the quantity of breastmilk, information was gathered on the frequency and length of breastfeeds.

**Results:**

At six months, 70% of infants were breastfed and 94% were given complementary foods. The average daily caloric intake was higher in non-breastfed (723 Kcal) than in breastfed infants (547 Kcal, p < 0.001) due to energy provided by complementary foods (321 vs. 190 Kcal, p < 0.001) and milk (363 vs. 301 Kcal, p = 0.007). Non-breastfed infants had also higher intakes of carbohydrates, proteins, and fats. The mean intake of macronutrients was within recommended ranges in both groups, except for the higher protein intake in non-breastfed infants. These consumed significantly higher quantities of commercial baby foods than breastfed infants.

**Conclusions:**

Contrary to what is recommended, 94% of infants were not exclusively breastfed and were given complementary foods at six months. The proportion of daily energy intake from complementary foods was around 50% higher than recommended and with significant differences between breastfed and non-breastfed infants, with possible consequences for future nutrition and health.

## Background

Adequate nutrition during the period of complementary feeding, from the time when other foods and/or drinks are added to a breastmilk- and/or formula-only diet to the time when breast- and/or formula-feeding is discontinued, is recognized as an important predictor of health later in life [1-3]. The World Health Organization (WHO) [4], the Italian Ministry of Health [5], several other European governments, and many professional associations, such as the American Academy of Pediatrics [6], recommend, as a public health measure, that infants be exclusively breastfed for the first six months of life to achieve optimal growth, health and development. In the course of the following six months, on average, the infant’s diet should gradually move towards a healthy family diet, without restrictions on the type of food, except for the replacement of breastmilk and/or formula with cow milk, which is not recommended before 12 months of age [7]. Contrary to the WHO recommendation, the European Society for Pediatric Gastroenterology Hepatology and Nutrition (ESPGHAN) recommends the introduction of complementary foods between 17 and 26 weeks of age [8]. Besides stirring up the scientific debate [9], statements such as this confuse parents and health professionals. Because of conflicting advice, but also for a series of cultural, social and economic reasons, exclusive breastfeeding up to six months falls short of recommended rates at global, regional, national and local levels [10-12]. Despite a very high rate of initiation of breastfeeding, for example, only 6% of infants are exclusively breastfed at six months in the Northeast of Italy [13]. The introduction of complementary foods is usually started between the ages of 4 and 5.9 months [14]. The first foods given to most infants, in Europe and in Italy, are fruit and cereals [15,16]. However, there is very little literature describing complementary feeding practices and nutrients’ intake in detail during this period [17-20]. The choice of certain first foods rather than others is probably linked to a presumed, and widely believed, association between certain food types and allergy; this association, however, is not based on evidence [8]. In addition, since many first complementary foods are available as commercial products, parents often use these, as opposed to healthy family foods [21]. It is estimated that up to 50% of parents use commercial baby foods for weaning [22,23]. Although the European Commission regulates the nutrient and micronutrient contents of infant foods, some studies show that these products may contain toxic elements [24-27]. Moreover, their flavour is often perceived by infants as unfamiliar, which may lead to temporary rejection, as infants like flavours they already know because of experience during pregnancy and lactation [28,29]. For these reasons, the use of commercial baby foods could make the acceptance of the family diet more difficult. The objective of our study was to describe in detail the feeding practices of a cohort of children from birth to 36 month. The patterns of breastfeeding and the timing of introduction of other foods and fluids, including infant formula, have already been described in a previous paper [13]. In this paper, we present detailed data on nutrients’ intake at six month of age and we investigate the association between feeding practices and breast- or formula-feeding, as well as the use of commercial baby foods.

## Methods

The cohort was recruited at the Institute for Maternal and Child Health, the only maternity hospital in Trieste, Italy, between July 2007 and July 2008, and followed up for three years through telephone interviews and self reported diaries to investigate the feeding practices of infants and the attitudes of mothers. The study was approved by the ethics committee of the Institute. The study design, protocol and sampling procedures have been previously described [13]. In brief, a cohort of 400 mother and infants pairs were enrolled at birth using the following eligibility criteria: birth weight ≥ 2000 g, no congenital malformation nor severe diseases that required hospital admission, gestational age of 36 completed weeks or more, and mothers’ residence in the province of Trieste. Upon enrolment, mothers were checked for eligibility and asked to give their informed consent. During the first contact, mothers were given a feeding diary with instructions on how to record type, quantity, and method of feeding over a 24-hour period on three separate non consecutive days, including one at the weekend, at 3, 6, 9, 12, 18, 24 and 36 months of age of the infant. Feeding diaries included a table with estimates in grams of common measures (e.g., teaspoon, cup) [30]. Mothers were asked to validate these estimates by actually weighing the amounts of their measures before using the diary the first time. Measures recorded by mothers (e.g., half a teaspoon) were then translated into grams for the database. Mothers were instructed to report salt and spices as pinches; these were translated into grams as validated by a previous Italian study [31].

Mothers were asked to report weight and length of their children as measured by their paediatrician during periodic health checks at 1, 3, 5–6, 8, 12, 18, 24 and 36 months of age. The range for inclusion of data in the analysis was ±15 days at 3 and 6 months, ±30 days at 9 and 12 months, and ±45 days at 18, 24 and 36 months of age. Additional demographic, educational, social, and anthropometric data on the mother, the father and the infant were obtained from a questionnaire.

The scope of this paper is limited to describing nutrients’ intake at six months. At this time, mothers were also asked to fill in a questionnaire on their sources of information about complementary feeding. Data extracted from the feeding diaries were analysed with Microdiet software (V2.8.6, Downlee Systems Ltd., High, Peak, UK). The nutrient analysis was carried out using the Italian food composition database that was included in the software [32]. For foods not included in the database, the Italian food composition database was updated using, whenever possible, the food labels of commercial products or making an estimate from composite foods using reported (e.g. cream peas with cereal flour) or standard recipes [30]. If the amount of a nutrient was not available, due to its absence in the food composition database or in the food label, this was considered as a missing value. There were no missing values for total protein, fat and carbohydrates; missing values for other nutrients ranged between 3% (sodium) and 96% (vitamin B12), with most values for all other nutrients around 20%. Only 0.5% of missing values were due to the food composition database; all the remaining missing values were due to lack of information in commercial food labels. The method used to estimate the food sources of single nutrients was based on the one proposed by Noble and Emmett [18].

To compare the nutrients’ intake between breastfed and non breastfed infants, mothers were split into a breastfeeding (ABF = any breastfeeding + formula + complementary feeding) and a non breastfeeding (NBF = formula feeding or cow milk + complementary feeding) group. To estimate the quantity of breastmilk, information on the frequency of breastfeeding combined with an estimated length of each feed was recorded in the diary [33]. Depending on whether the length of the feed estimated by the mother was described as “short”, “normal” or “long”, volumes of 60, 80 and 100 ml, respectively, were assigned to that feed. The nutritional content of breastmilk was calculated according to published values [34,35]. Although this method only provides an estimate of the quantity of breastmilk consumed, it is the method used in other studies and the results we obtained are comparable [19].

For the analysis of the results, complementary foods were classified in food groups (milk and milk products; cereals; meat; fish; pulses; eggs; fruit; vegetables; nuts and seeds; fats and oils; sugars; sweets and desserts; soft drinks; tubers; herbs and spices; sauces; cured meats) and in four food types (commercial baby foods; non-commercial baby foods; breastmilk; formula). Continuous data are presented as median and interquartile range (IQR) or as mean and standard deviation (sd). Anthropometric measures were compared with the WHO growth standards using WHO Anthro software, and are reported as body mass index (BMI) z-score [36]. Categorical data are presented as absolute frequencies and percentages. Differences in continuous variables were assessed using Mann–Whitney test or t-test as appropriate, while differences between categorical variables were analysed with Fisher exact test.

## Results

**Figure 1:**
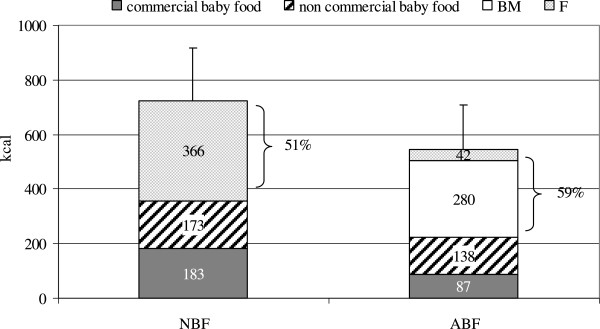
Daily caloric intake (Kcal) from breastmilk (BM), formula (F), commercial and non commercial baby food in ABF (n = 188) and NBF (n = 80) infants (n = 268).

Four hundred mother-and-child pairs were enrolled in the study; complete data from the six months follow up are available for 268 (67%). Mothers had a mean age of 33.4 years (sd 4.5), 89% had a medium-to-high level of education, and 81% were employed. These values were similar in the ABF and NBF groups and there were no statistically significant differences. The percentage of employed mothers was lower at six months (81%) than at enrolment (95%) [13], probably because some mothers with a temporary job had meanwhile become unemployed. Employment status, however, was not associated with the initiation nor with the continuation of breastfeeding. Most infants (91%) were born between 38 and 42 weeks of gestation and 80% had a vaginal delivery. Seventeen (5%) infants weighed more than 4,200 g at birth, 54 (16%) were over 53 cm long, these values corresponding to the 97^th^ WHO percentile [36]. Additional details collected at enrolment have already been published [13]. Mothers lost to follow up had a higher level of education and were more likely to be Italian and to be employed at baseline compared to those still in the study at six months, but there were no significant differences in breastfeeding rates at enrolment. At six months, most infants were healthy, except for 10% (26/268) affected by minor ailments (flu or cold) one or more days during the period of data collection. Anthropometric data of 185 out of 268 infants were collected at the routine well-child visits and are shown in Table 1. The mean BMI z-score was −0.36 (sd 1.0), slightly lower than the WHO standard [36], with no statistically significant differences between ABF and NBF infants (p = 0.206). There were no significant differences in energy and nutrients intakes between infants with or without anthropometric data.At six months of age, 70% (188/268) of infants were still breastfed; 94% (252/268) were receiving complementary foods with no statistically significant differences between ABF and NBF infants (93% vs. 96%, p = 0.407). The mean daily caloric intake was 599 Kcal and Figure 1 shows the breakdown by food category and the differences between groups. The overall daily caloric intake was higher in NBF (mean 723 Kcal, sd 194; median 680 Kcal, IQR 541–849) than in ABF infants (mean 547 Kcal, sd 162; median 528 Kcal, IQR 443–645) (p < 0.001) due to the energy provided by complementary food (median 321 Kcal, IQR 157–526 vs. median 190 Kcal, IQR 56–356; p < 0.001) and milk (median 363 Kcal, IQR 274–469 vs. 301 Kcal, IQR 243–393; p = 0.007). The mean energy density of complementary foods was higher in NBF than in ABF infants: 0.97 vs. 0.86 Kcal/g (p = 0.015). NBF infants had a slightly higher intake of milk (formula or formula + cow milk) than ABF infants (estimated breastmilk only or estimated breastmilk + formula), but differences were not statistically significant (median 545 g, IQR 420–700 vs. 485 g, IQR 393–640; p = 0.145).

**Table 1:** Characteristics of infants at six months

	**All infants**		**ABF**		**NBF**		**p value**
	**Mean (n)**	**sd**	**Mean (n)**	**sd**	**Mean (n)**	**sd**	
Age (months)	5.7 (185)	1.0	5.6 (137)	1.1	5.8 (48)	0.5	0.981
Weight (kg)	7.6 (186)	0.9	7.5 (138)	0.1	7.8 (48)	1.1	0.065
Length (cm)	67.3 (184)	2.5	67.2 (137)	0.2	67.8 (47)	2.9	0.131
BMI Z-score	−0.36 (181)	1.0	−0.4 (134)	1.1	−0.2 (47)	1.0	0.206
Solids introduced	% (n)		% (n)		% (n)		
Yes	94 (252)		93 (175)		96 (77)		0.407
No	6 (16)		7 (13)		4 (3)		

NBF infants had a higher overall intake of carbohydrates (except for simple sugars), proteins and fats than ABF infants (Table 2). Despite these differences, the overall mean intake of these macronutrients was within the recommended range in both groups, except for the higher protein intake in the NBF group [7]. The two groups differed for intake of micronutrients and monounsaturated and saturated fats, the intakes in NBF infants being higher than in ABF infants and higher than recommended [7]. The intakes of vitamins D and A were lower than recommended in both groups: in NBF infants they covered 70% and 89% of recommended intakes, respectively; in ABF infants 10% and 91%. In the ABF group calcium and niacin intakes were also lower than recommended, 61% and 63% of recommended intakes, respectively. The intake of cholesterol in ABF infants was four times higher than in NBF infants.

**Table 2:** Daily intake of nutrients (percentage of energy or mean quantity of three days)

**Nutrient**	**All infants**		**ABF**		**NBF**		**p value**	**Recommended daily intake**^**a**^
	**(n = 268)**		**(n = 188)**		**(n = 80)**			
	**Mean**	**sd**	**Mean**	**sd**	**Mean**	**sd**		
Protein (%)	10%		10%		11%			
Protein (g)	15	8	13	7	20	8	<0.001	14 g
Total CHO (%)	52%		51%		54%			55-65%
Sugars (%)	32%		34%		27%			
Total CHO (g)	79	28	69	23	97	30	<0.001	
Sugars (g)	48	13	47	13	49	14	0.278	
Fat (%)	38%		39%		35%			30-40%
Fat (g)	25	7	24	7	29	9	<0.001	
Sfa (g)	9	3	9	3	9	3	0.736	
Mufa (g)	10	4	9	3	12	5	<0.001	
Pufa (g)	3	1	3	1	4	2	<0.001	
Cholesterol (mg)	68	42	83	33	20	27	<0.001	
Fibre (g)	5	4	4	4	6	4	<0.001	
Starch (g)	15	13	12	12	19	15	<0.001	
Sodium (mg)	373	321	338	297	455	362	<0.001	
Potassium (mg)	747	408	693	391	875	422	<0.001	
Calcium (mg)	383	184	307	130	562	168	<0.001	500 mg
Zinc (mg)	4	1	3	1	5	1	<0.001	3.1 mg
Iron (mg)	4	4	2	2	7	6	<0.001	
Vitamin B1 (mg)	0.5	0.4	0.4	0.4	0.8	0.2	<0.001	0.3 mg
Vitamin B2 (mg)	0.7	0.5	0.5	0.4	1	0.3	<0.001	0.5 mg
Vitamin B6 (mg)	0.5	0.5	0.4	0.5	0.8	0.3	<0.001	
Vitamin B12 (μg)	0.6	1.01	0.4	0.4	1.2	2	<0.001	
Vitamin C (mg)	63	32	51	26	92	28	<0.001	20 mg
Vitamin D (μg)	3	3	1	2	7	2	<0.001	10 μg
Vitamin E (mg)	1.5	1	1.5	1	1.6	1	0.631	1.14-15.2 mg^b^
Vitamin A (μg)	315	374	317	390	310	339	0.701	350 μg
Niacin (mg)	4.4	5	3.4	6	6.8	2	<0.001	5.4 mg
Folate (μg)	98	48	87	47	122	42	<0.001	24 μg
Oleic acid (g)	9	4	9	3	11	5	<0.001	
Linoleic acid (g)	3	1	2	1	4	1	<0.001	2 g^c^
Linolenic acid (g)	1.1	1	0.8	1	1.6	0.5	<0.001	0.2 g^c^

^a^[7].

^b^The recommended daily intake of vitamin E for infants 4–6 months old is 0.15-2 mg/kg. The values shown in the table are based on the mean weight of 7.6 kg (n = 207).

^c^The recommended daily intakes of linoleic and linolenic acid for 6-months old infants are 3% and 0.3% of total energy intake, respectively. The values shown in the table are based on the mean energy intake of 599 Kcal (n = 268).

**Figure 2:**
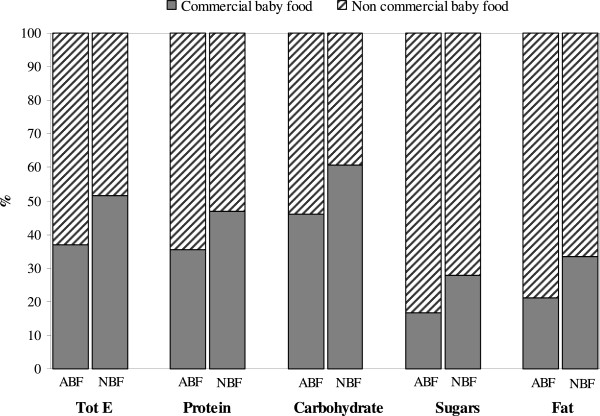
Total energy (Tot E) and macronutrient intake (%) from complementary foods only in ABF (n = 175) and NBF (n = 77) infants (n = 252) by commercial and non commercial foods; all differences are significant (p < 0.001).

Complementary feeding was analysed distinguishing home made from commercial baby food and identifying the contribution of each of these in terms of nutrient intake. Overall, home-made food contributed more nutrients in both ABF and NBF infants. NBF infants consumed significantly higher quantities of commercial baby foods than ABF infants, with a consequent higher intake of macronutrients from these foods (p < 0.001). In particular, commercial baby foods represented the main source of carbohydrates in NBF infants (Figure 2). At six months, infants were given fruit (92%), cereals (76%), vegetables (72%), oils and fats (68%), meat (55%), sweets and desserts (45%), and tubers (30%), in addition to breastmilk, formula or cow-milk. Less than 10% were given fish, pulses and cured meat. Only one infant was given an egg, another one nuts, both in the NBF group. Two infants were given sauces, one in the ABF and one in the NBF group, with an irrelevant contribution to nutrients and energy intakes. As far as drinks, in addition to milk and broth, are concerned, 19 infants were given sweetened drinks such as fruit juices and teas. Table 3 shows the percentage of consumption of each food group, its daily intake (g/d) and its caloric contribution (%). NBF infants ate significantly higher quantities of milk and milk products (584 vs. 508 g/d), fruit (93 vs. 69 g/d), and sweets and desserts (21 vs.11 g/d), while ABF infants received a significantly higher proportion of energy from milk and milk products (67% vs. 61%) and from oils and fats (6% vs. 4%), but less from sweets and desserts (6% vs. 8%).

**Table 3:** Number and percentage of infants eating selected foods and food groups, and relative daily intake (g/d)

**Food group**	**n**	**Daily intake (g/d)**					**Energy intake (%)**
		**All infants (n = 268)**	**ABF (n = 188)**		**NBF (n = 80)**		**All infants (n = 268)**
		**Median (IQR)**	**n (%)**	**Median (IQR)**	**n (%)**	**Median (IQR)**	**Median (IQR)**
Cereals	193	17.8 (8–33)	128 (68)^a^	17 (8–27)	65 (81)^a^	24 (9–40)	11 (5–17)
Milk, milk products	268	547 (438–693)	188 (100)	508 (429–643)^a^	80 (100)	584 (487–719)^a^	65 (50–85)^b^
Vegetables	181	76 (38–137)	127 (68)	73 (37–133)	54 (68)	80 (40–142)	4 (2–7)
Fruits	232	75 (37–122)	161 (86)	69 (30–116)^a^	71 (89)	93 (48–142)^a^	8 (4–13)
Spices, salt	14	1 (1–2)	11 (6)	2 (1–2)	3 (4)	1 (1–4)	0 (0–0)
Fish	26	17 (13–33)	17 (9)	17 (13–27)	9 (11)	13 (13–33)	2 (1–4)
Meat	140	26 (10–38)	89 (47)^a^	20 (10–35)	51 (64)^a^	27 (10–40)	3.5 (2–6)
Fat, oil	172	3 (2–6)	121 (64)	4 (2–6)	51 (64)	3 (2–7)	5 (4–9)^b^
Sweets, desserts	113	13 (7–37)	62 (33)^a^	11 (5–24)^a^	51 (64)^a^	21 (8–58)^a^	7 (4–12)^b^
Tubers	73	32 (14–49)	47 (25)	32 (17–53)	26 (33)	31 (10–49)	4 (2–6)
Broth	120	75 (33–133)	81 (44)	71 (27–124)	38 (48)	95 (46–147)	0.4 (0–1)
Sweet drinks	19	42 (17–100)	13 (7)	30 (17–77)	6 (8)	51 (33–130)	4 (2–8)
Pulses	11	24 (8–50)	5 (3)	13 (8–17)	6 (8)	42 (24–53)	2 (2–12)
Cured meat	14	13 (7–17)	8 (4)	13 (7–15)	6 (8)	13 (10–17)	2 (1–4)

^a^p < 0.05 for differences in percentage of infants or daily intakes between ABF and NBF.

^b^p < 0.05 for differences in percentage of energy intakes between ABF and NBF.

Mothers reported receiving the main information on infant feeding from health professionals, in particular from paediatricians (93%), from their own mother or friends (64%), and from the media, in particular magazines (60%). However, these sources of information appear not to be associated with the decision to give infants commercial or home-made foods (p > 0.05).

## Discussion

Contrary to the WHO recommendations and to the policy of the Italian Ministry of Health [4,5], 94% of infants in our cohort were not exclusively breastfed to six months and had already been given complementary foods by this age, at which only a smaller proportion of infants may be developmentally ready to introduce solids [9,37]. This finding is confirmed by a report that used data from a different source, the records kept by family paediatricians at well child visits in three regions of Italy, showing that 85% of infants had already been given complementary foods at six months [16]. This is probably the effect of the advice given by paediatricians as a result of the ESPGHAN recommendations [8]. Based on our results, the paediatricians were the main source of information for mothers. As a consequence, the proportion of daily energy intake from complementary foods at six months was around 50%, higher than recommended [7]. Instead of getting most of their energy and nutrients from breastmilk or formula, infants received them from solids, with significant differences between those who still received breastmilk, the ABF group, and those who did not, the NBF group (41% vs. 49%, p < 0.001). Also, adding the calories from complementary foods to those from milk, ABF infants consumed significantly less calories than NBF infants, and had consequently a lower intake of macronutrients, though always within the recommended levels [7]. As far as anthropometric measures at six months are concerned, ABF infants fared as well as NBF infants, despite their lower caloric intake. NBF infants, in addition, displayed a higher than recommended intake of proteins, as confirmed by other studies [17,19]. This point may be important, given the growing evidence that a high intake of proteins early on in life, though not necessarily at six months, may be associated with a higher risk of obesity later on [38,39].

The fat intake, particularly that of cholesterol, was proportionally higher in ABF than in NBF infants. Cholesterol, in fact, is present in high quantity in human milk and represents a very important factor for infant growth as it is the basic component of cell membranes [40]. NBF infants had higher than recommended intake of most micronutrients, except for vitamins A and D. On the contrary, ABF infants had lower than recommended intake of some micronutrients, as reported by other studies [19,20], in particular calcium and iron. The relative deficiency of these elements in their diet, however, may be balanced by their higher bioavailability in breastmilk [41-44], and by exposure to sunshine as far as vitamin D is concerned. It would be advisable, however, to include foods rich in calcium and iron as first complementary foods in breastfed infants.

ABF and NBF infants were also different in feeding choices. Breastfed infants consumed lower quantity of commercial baby food. Although the consumption of home-made food was prevalent in the diet of both groups of infants, it is interesting to note that the mean energy contribution per gram of commercial baby foods was higher than that of home-made foods (1.6 Kcal/g vs. 0.7 Kcal/g). If this is true, i.e. if commercial baby foods are more energy dense than non commercial products, great attention on their nutrient composition is needed when they are used as substitutes of home- made foods. Finally, the food group analysis shows that at six months the infant diet was characterized mainly by milk, cereals, fruit, vegetables and fats consumed mostly in mashed form like a porridge. This is confirmed also by a recent cross-sectional study [16], and may be associated with some delay in developmental readiness for solids and later acceptance of family foods [45].

To our knowledge, this is the first study in Italy that investigated in detail the differences in feeding practice, food and nutrient intake between breast- and formula-fed six months old infants. The study, however, has some limitations that must be taken into account when interpreting the results. The first limitation is the loss to follow up between birth and six months, probably due to the length and complexity of the questionnaires and diaries used to collect feeding data. In spite of this loss and of some differences with the initial sample, we ended up with 268 subjects, a sample size comparable to those analysed by other reports of this kind. The second limitation is that anthropometric data were not gathered by the study team; this would have duplicated a practice already carried out by family paediatricians and would have added a further burden to the mothers. As a consequence of this lack of direct control, anthropometric measures were reported only by 185 mothers and data were not collected using standard methods. This may bias the results, despite the fact that there were no significant differences in energy and nutrients intakes between infants with or without anthropometric data. Third, mothers may have misreported real food intake in their diaries; this is more likely to occur with repeated dietary assessments in the same subjects [46]. In this paper, however, we report the results of a single and early set of feeding diaries, unlikely to be much affected by misreporting. Fourth, the consumption of breastmilk was estimated based on the frequency of feeds and the perceived length of each feed, and taking into account the average composition of human milk. A small underestimation of the intake of breastmilk may have occurred; the overall daily intake of milk, however, does not show significant differences when comparing NBF (formula or formula + cow milk) with ABF (estimated breastmilk only or estimated breastmilk + formula) infants. The mean energy intake of ABF infants was 547 kcal/d, a value lower than the one estimated by WHO [7], but comparable to the one from a recent study [47]. Finally, the assessment of the intake of some micronutrients was incomplete due to the lack of information on these elements in the labels of commercial baby food which prevented a complete nutritional analysis for infants consuming these products.

## Conclusions

To conclude, our study provides a detailed examination of the feeding practices of a sample of Italian six months old infants during the period of complementary feeding. Our results show that breastfed infants receive less calories, less food, and consume less commercial baby foods than not breastfed infants. Our findings confirm the need for special support for formula fed infants in order to avoid excessive nutrient intake. They also confirm the need to enhance activities for the protection and support of exclusive breastfeeding, as recommended by WHO and the Italian Ministry of Health. Finally, our results point to the need to avoid that conflicting and confusing recommendations and advice regarding the timing of introduction of solids be given to parents.

## Abbreviations

ABF: Any breastfeeding + formula + complementary feeding; BMI: Body mass index; CHO: Carbohydrates; ESPGHAN: European Society for Pediatric Gastroenterology Hepatology and Nutrition; IQR: Interquartile range; Mufa: Monounsaturated fatty acids; NBF: Formula feeding or cow milk + complementary feeding; Pufa: Polyunsaturated fatty acids; Sfa: Saturated fatty acids; WHO: World Health Organization.

## Competing interests

The authors declare that they have no competing interests.

## Authors’ contributions

AC designed and supervised the study, helped analyse and interpret the results, and revised the manuscript. PP, CC and AK conducted the study, carried out the interviews, constructed the database, helped analyse and interpret the results, and wrote the manuscript. MP and FC derived nutrient intakes from the database, and helped analyse and interpret the results. MM was in charge of the statistical analysis. All authors read and approved the final manuscript.

## Pre-publication history

The pre-publication history for this paper can be accessed here:

http://www.biomedcentral.com/1471-2431/14/127/prepub
